# Poor neuro-motor tuning of the human larynx: a comparison of sung and whistled pitch imitation

**DOI:** 10.1098/rsos.171544

**Published:** 2018-04-18

**Authors:** Michel Belyk, Joseph F. Johnson, Sonja A. Kotz

**Affiliations:** 1 Bloorview Research Institute, 150 Kilgour Road, Toronto, Canada M4G 1R8; 2 Faculty of Psychology and Neuroscience, University of Maastricht, Maastricht, The Netherlands; 3 Department of Neuropsychology, Max Planck Institute for Human and Cognitive Sciences, Leipzig, Germany

**Keywords:** imitation, voice, larynx, articulation, motor control, evolution

## Abstract

Vocal imitation is a hallmark of human communication that underlies the capacity to learn to speak and sing. Even so, poor vocal imitation abilities are surprisingly common in the general population and even expert vocalists cannot match the precision of a musical instrument. Although humans have evolved a greater degree of control over the laryngeal muscles that govern voice production, this ability may be underdeveloped compared with control over the articulatory muscles, such as the tongue and lips, volitional control of which emerged earlier in primate evolution. Human participants imitated simple melodies by either singing (i.e. producing pitch with the larynx) or whistling (i.e. producing pitch with the lips and tongue). Sung notes were systematically biased towards each individual's habitual pitch, which we hypothesize may act to conserve muscular effort. Furthermore, while participants who sung more precisely also whistled more precisely, sung imitations were less precise than whistled imitations. The laryngeal muscles that control voice production are under less precise control than the oral muscles that are involved in whistling. This imprecision may be due to the relatively recent evolution of volitional laryngeal-motor control in humans, which may be tuned just well enough for the coarse modulation of vocal-pitch in speech.

## 1. Introduction

Vocal imitation is a hallmark of human communication that underlies the capacity to learn to speak and sing. It is the ability to reproduce previously experienced auditory events by computing an inverse model that maps target sounds onto motor commands that reproduce them [1]. Whereas the ability to flexibly produce calls from an existing repertoire (vocal usage learning) is relatively common, the ability to add new vocalizations to an existing repertoire (vocal production learning) is rare in mammals [2,3]. Among the principal exceptions are humans, cetaceans [4–6], pinnipeds [7–11], bats [12,13] and possibly elephants [14,15]. How the hominid vocal phenotype evolved from vocal usage to vocal production learning remains a matter of speculation. Several plausible hypotheses have been advanced to suggest, for example, that emotional expression may have provided a scaffold for the evolution of speech and song [16], or that the exaggeration of social dominance cues provided selective pressure for more versatile voices [17], which may have been exploited by early hominins for the purpose of communication [18].

Vocalizations are composed of a periodic signal produced by the larynx that is filtered depending on the configuration of the vocal tract, such as the articulation of the lips and tongue [19–21]. This system of sound source and filter is a useful bioacoustic description and provides a framework for understanding the muscles of communication and the evolution of volitional control over them. For example, research has increasingly come to suggest that non-human great apes may have more flexible call repertoires than previously supposed. This includes a limited degree of flexibility at the laryngeal sound source [22–24], but much more extensive control over the shape of the vocal tract via movement of the lips and tongue. Indeed, great apes have been observed to learn to produce a variety of non-species typical oral sounds such as raspberries and whistles that involve the lips and tongue instead of the larynx [25–27]. Similarities between human speech and great ape lip-smacking behaviours [28–30], as well as in the range and flexibility of tongue movements in these species [31,32], suggest that the orofacial articulatory muscles were speech-ready in ancestral primates. By contrast, humans have a clear advantage over other primates in controlling the laryngeal sound source as we modulate vocal-pitch not only to sing, but also to encode voiced compared to voiceless phonemes [33,34], the tones of tonal languages [35], stress on particular syllables [36], emphasis on certain words [37], the intonation of sentences to contrast declarative and interrogative modes [38], and to express a broad range of genuine or feigned emotions [39–43].

Despite the vocal virtuosity of humans relative to other apes, there is a population of individuals—colloquially referred to as ‘tone deaf’—who are notable in their poor abilities as singers. However, tone deafness is a misnomer, as these individuals do not necessarily have a deficit in hearing musical sounds, but rather in singing them [44–46]. These individuals are more accurately described as poor-pitch singers, as they appear to have a selective deficit in translating perceived pitches into the sequence of laryngeal-motor commands that reproduce them [47]. In some cases this results in inaccurate singing—that is consistently flat or consistently sharp—but more often it manifests as imprecise singing—that is highly variable [48]. Rather than a discrete population, poor-pitch singers appear to be the low proficiency tail of a continuous range of singing abilities [45,49,50].

Even professional opera singers, who presumably occupy the high proficiency tail of the singing proficiency continuum, are less precise with vocal-pitch when singing than violinists are with the pitch of their instruments [51,52] and may be unreliable judges of whether they themselves have just produced an error [53]. A lifetime of experience with the imprecision of the voice may explain why listeners are more generous in judging whether a vocalist is in tune than when judging an instrumentalist [54]. Across levels of training, singers match pitches more accurately with an instrument than with their voices, despite unfamiliarity with the instrument [55,56]. This pattern holds even with digital instruments that produce a vocal timbre [57,58], suggesting that poor pitch matching is rooted in vocal motor-control rather than deficient perception of vocal-pitch.

This lack of vocal proficiency is striking in humans, who are the most vocally proficient species of ape. Though there are neuro-comparative differences between humans and other primates in several brain areas related to the control of the vocal tract [59–62], one of the more striking comparative differences is specific to the laryngeal muscles that control the voice. Humans possess a direct pathway projecting from the larynx-motor cortex to the nucleus ambiguus, which is the brainstem-motor nucleus that controls the laryngeal muscles [63,64]. This direct pathway is less abundant in other great apes [65] and absent in monkeys [66,67]. However, even in humans this pathway remains sparse compared to the analogous pathways that descend to the brainstem-motor nuclei that control the muscles of the lip and tongue [63–65].

These observations lead us to hypothesize that the human vocal-motor system is not tuned as precisely as other orofacial neuro-motor systems. To test whether humans are imprecise singers, we had participants listen to and then imitate simple melodies by either singing or whistling. These tasks were highly matched in auditory and cognitive demands, differing only in whether pitches were imitated by vocalizing the neutral vowel schwa or by producing a bilabial whistle. We hypothesized that imitation errors would tend to be larger for singing than for whistling.

## 2. Methods

### 2.1. Stimulus generation

Two sets of 45 melodies were composed by random computerized composition. The first note of each melody was selected at random from a chromatic scale. Subsequent notes were determined by sampling from a flat distribution of interval sizes, ranging ±4 semitone. This process was performed iteratively until all notes fell within the range of a single octave. All melodies consisted of a sequence of five isochronous notes each lasting 750 ms separated by 50 ms of silence. Both sets of melodies were synthesized in a vocal timbre using a neutral vowel (Vocaloid Miriam, Zero-G Limited, Okehampton, UK) as well as in a timbre that approximates a human bilabial whistle (see electronic supplementary material, S1 and S2). All stimuli had a sampling rate of 44 100 Hz with 16-bit digitization of amplitudes. Two versions of the vocal-timbre stimuli were synthesized to accommodate the disparate vocal ranges of males and females. These spanned the range A2–A3 (110–220 Hz) and A3–A4 (220–440 Hz) for males and females, respectively. The whistled-timbre stimuli were synthesized in the range A5–A6 (880–1760 Hz). Whistled-timbre stimuli were synthesized as a sine wave convolved with an empirical estimate of the onset amplitude envelope observed in pilot experiments. The pitch ranges and the onset amplitude envelope associated with the timbre of a bilabial whistle were estimated from 30 recordings taken from five males and five females. These recordings were collected during pilot experiments in which participants performed the individual assessment of producible range of pitches described below. All stimuli were synthesized at equal sound pressure levels.

### 2.2. Procedures

#### 2.2.1. Participants

Thirty-four participants were recruited through two separate listings in the undergraduate testing pool of the Faculty of Psychology and Neuroscience at Maastricht University. The two listings were worded to attract either strong or poor singers in order to draw from both ends of the spectrum of singing ability, but made no reference to whistling to avoid sampling bias. Six participants were unable to produce any pitched sound by whistling. Only data from the remaining 28 participants were analysed. These participants had a median age of 21 years (range 18–29), 20 were female, nine self-identified as a good singer, 15 self-identified as a good whistler, 26 had some degree of formal musical training (2–15 years) but only two of these had any vocal training. All participants reported normal hearing and no vocal pathology. All participants provided informed consent and were compensated with either course credit or a 10€ voucher.

#### 2.2.2. Individual assessment of producible frequency ranges

Recordings were performed in a sound-attenuated chamber using a desk-mounted Sennheiser microphone and Adobe Audition software (v. 1.5). Participants were instructed to sing (i) a stable and comfortable note, (ii) a descending sweep as a smoothly varying pitch contour from a comfortable note to their lowest producible note, and (iii) an ascending sweep from a comfortable note to their highest producible note. Each production task was repeated three times. The mean frequency of the comfortable note was measured using Praat (v. 6.0.17; www.fon.hum.uva.nl/praat/) and taken as each participant's habitual pitch. The highest and lowest frequencies produced during vocal sweeps were used to estimate each participant's producible range. The same procedure was repeated for whistling.

#### 2.2.3. Imitation task

In the same recording environment, participants performed two audio-motor imitation tasks: once imitating one set of melodies presented in a vocal timbre and sex-appropriate vocal range by singing, and once imitating a second set of melodies presented in a whistled timbre by whistling. Participants were instructed to sing using only a neutral vowel that was also the carrier vowel of the stimulus. Each task consisted of listening to and then repeating 45 melodies consisting of five notes each. Each melody was presented one at a time and separated by 7 s silent gaps during which participants' imitations were recorded. Stimulus onset times were jittered by 250, 500 or 750 ms. Participants were given the opportunity to rest for a duration of their choosing after every 15th trial. Both the order of imitation conditions and the sets of target melodies were counter-balanced across participants. Melodies were presented in random order within conditions. Stimulus presentation and sound recordings were managed through Python (v. 2.7; python.org). Target stimuli were played over free field speakers at a comfortable volume.

#### 2.2.4. Melodic discrimination

We assessed participants’ ability to perceive pitches within a melodic context and retain them in working memory using a computerized version of the Montreal Battery of Amusia Evaluation (MBEA) [68] programmed in Python. Stimuli were presented over free field speakers while participants were seated alone in a sound attenuated booth. Only the subscales of the MBEA that assess pitch perception (1a–1c) were completed. Participants listened to three sets of 30 pairs of melodies that were either identical or had one note transposed, and indicated whether the melodies were the same or different. Each set contained transpositions that were increasingly difficult to detect and each set was preceded by two practice trials.

### 2.3. Acoustic analysis

An in-house Praat script was used to semi-automate the extraction of fundamental frequency (*F*_0_) from the centre 250 ms of each imitated note. This script is available in the online data supplement to this article (http://dx.doi.org/10.5061/dryad.504t7 [69]). Melodies that were produced with too few or too many notes were excluded from further analysis because the positions of omitted or duplicated notes were not possible to determine (totalling 3.6% of trials). Responses to stimuli that were outside of each participant's producible range were excluded as they may reflect limitations of the producible range rather than imitation ability.

*F*_0_ values were converted from hertz to cents relative to lowest scale degree of the stimulus set, where 100 cents is equal to one semitone and 1200 cents is equal to one octave of the equal temperament scale (equation (2.1)). Note error was calculated as the differences between the pitches of the target melody and participants' imitations. Intervals are the difference between adjacent notes in a melody. Interval error was calculated as the differences between intervals in the target melody and intervals in participants’ imitations. All produced notes associated with errors greater than 1000 cents were verified for measurement errors, including octave errors.

2.1 $$\log_{2}\left(\frac{A}{B}\right)\times 1200.$$

We applied the approach of Pfordresher *et al*. [48] in separately calculating the accuracy and precision of imitated melodies. Inaccuracy reflects a consistent bias to produce responses that err in the same direction, for example, by consistently singing flat. Imprecision reflects the variability across repeated attempts to produce the same pitch, for example, by intermittently singing responses that are flat and sharp by varying degrees. Inaccuracy scores were calculated for each participant as the mean signed difference between target notes or intervals and imitated notes or intervals. Imprecision scores were calculated for each participant by finding the standard deviation of differences between the target and imitated notes or intervals within each pitch class, and taking the average across pitch classes.

## 3. Results

MBEA scores varied widely along a continuous range from 71% to 100% correct responses (mean 86.4%, s.d. 9.6%). The scores of three participants were below the conventional cut-off suggested to identify individuals with amusia [68]. The continuous range of scores observed in this sample and reported by Peretz *et al*. lead us to retain the data from all participants but include MBEA scores as a continuous predictor in subsequent analyses.

**Figure 1.**
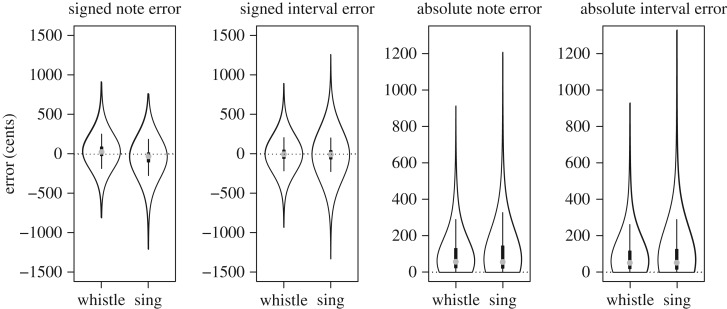
Violin plots. The four panels show box plots surrounded by density distributions of pitch errors for all notes produced by all participants. The two leftmost panels show signed errors and demonstrate that there is a small tendency for imitated notes to be sung flat or whistled sharp. The rightmost panels demonstrate that imitation errors while singing were much more variable than imitation errors while whistling.

Figure 1 shows violin plots of imitation errors across 11 433 notes produced by participants in this experiment and demonstrates a clear violation of heterogeneity of variance because singing appears to be more variable than whistling. We chose not to model these data using nonlinear regression techniques that are robust to heteroscedasticity because the systematic difference in variability between conditions is of theoretical interest. As an alternative, we computed (in)accuracy and (im)precision scores for each participant [48]. These scores passed all tests of assumptions for linear mixed models (LMMs).

We constructed four sets of nested LMMs to predict note inaccuracy, interval inaccuracy, note imprecision and interval imprecision from the two modalities of imitation and from MBEA scores, with participant modelled as a random intercept [70,71]. The effects of pitch-production modality and perceptual ability were tested by comparing nested LMMs; degrees of freedom and *p*-values were calculated using the Kenward–Roger approximation [72]. We report standardized estimates calculated by refitting each statistical model with input variables centred and scaled by 2 s.d. [73,74]. These effect sizes should be interpreted as the expected differences in outcome for levels of the predictor variables that are 1 s.d. below the mean compared to 1 s.d. above the mean [75]. For categorical predictors (such as whistling versus singing), this is equivalent to the estimated difference between conditions. For continuous predictors (such as MBEA score), this is equivalent to the difference in outcomes for participants with scores 1 s.d. below the mean (a score of 76%) to 1 s.d. above the mean (a score of 96%). Confidence intervals (CIs) for these estimates were determined by bootstrapping with 1000 iterations.

### 3.1. Imprecision

**Figure 2.**
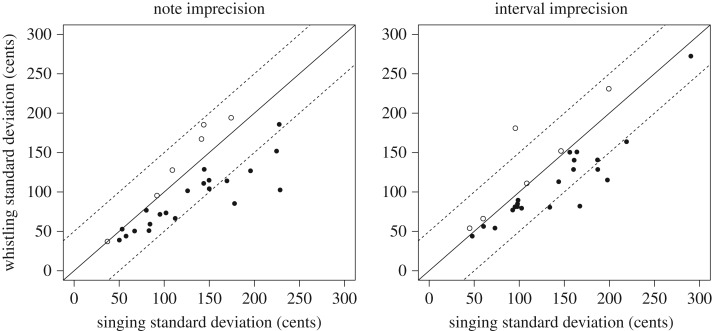
Imprecision. Singing imprecision is plotted on the *x*-axis and whistling imprecision is plotted on the *y*-axis for each participant. Larger scores indicate less precise imitation. The solid line indicates a hypothetical one-to-one correspondence between singing and whistling imprecision scores. Of 28 participants, 22 were below this line (filled circles) indicating that they were less precise when singing than whistling. Dashed lines indicate ±50 cents from the equal performance line, which is a conventional threshold for poor imitation.

Note imprecisions (*F*_1,25.3_ = 12.02, *p* < 0.05, standardized estimate = −25.5, 95% CI = −38.4 to −11.3) and interval imprecisions (*F*_1,25.2_ = 6.14, *p* < 0.05, standardized estimate = −17.0, 95% CI = −30.4 to −3.5) were both significantly higher for singing than whistling (figure 2). Both note imprecision (*F*_1,26.2_ = 36.92, *p* < 0.05, standardized estimate = −83.6, 95% CI = −112.3 to −54.9) and interval imprecision (*F*_1,25.8_ = 6.14, *p* < 0.05, standardized estimate = −92.4, 95% CI = −127.6 to −61.1) were significantly predicted by perceptual ability.

Figure 2 also highlights a strong relationship between singing and whistling precision scores. However, in the light of the common influence of perceptual ability on both of these scores we conducted partial correlations between singing and whistling scores, controlling for perceptual ability as estimated by the pitch subscales of the MBEA. There were significant partial correlations between singing and whistling scores for both note imprecision (*r*^2^ = 0.29, *p* < 0.05) and interval imprecision (*r*^2^ = 0.50, *p* < 0.05), demonstrating that the relationship between singing and whistling imprecision scores is not solely due to the common influence of perceptual ability.

### 3.2. Inaccuracy

**Figure 3.**
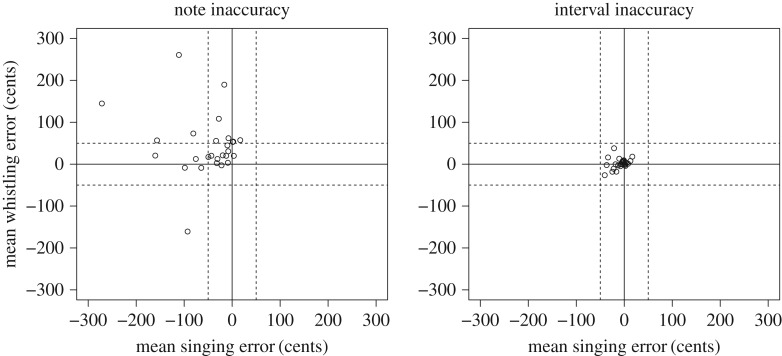
Inaccuracy. Singing inaccuracy is plotted on the *x*-axis and whistling inaccuracy is plotted on the *y*-axis for each participant. More extreme scores indicate less accurate imitation. Negative values indicate an average imitation that is flat and positive values indicate an average imitation that is sharp. Solid horizontal and vertical lines indicate inaccuracy scores of 0, or perfect performance. Dashed lines indicate ±50 cents as a conventional threshold for poor performance. Participants with scores beyond the dashed lines produced imitations that were closer to an out of tune note than to the target note. The notable disparity between participants note inaccuracy and interval inaccuracy scores may be explained by transposition. Participants appear to have consistently sung entire melodies up to 300 cents lower, or whistled melodies up to 200 cents higher, than the target melodies while retaining the correct relationship between notes within melodies.

Figure 3 highlights an overall tendency for sung notes to be flat and whistled notes to be sharp. Note inaccuracy (*F*_1,25.7_ = 27.03 *p* < 0.05, standardized estimate = 100.8, 95% CI = 6.5 to 138.8) and interval inaccuracy (*F*_1,25.5_ = 8.86, *p* < 0.05, standardized estimate = 9.2, 95% CI = 2.5 to 15.5) scores were significantly lower for singing compared to whistling. Neither note inaccuracy (*F*_1,26.8_ = 2.47, *p* = 0.13, standardized estimate = 41.9, 95% CI = −9.5 to 71.5) or interval inaccuracy (*F*_1,26.6_ = 0.64.0, *p* = 0.43, standardized estimate = 3.6, 95% CI = −4.9 to 11.9) were significantly predicted by perceptual ability. There was no significant partial correlation between singing and whistling scores for either note inaccuracy (*r*^2^ = 0.08, *p* = 0.15) or interval inaccuracy (*r*^2^ = 0.12, *p* = 0.07).

**Figure 4.**
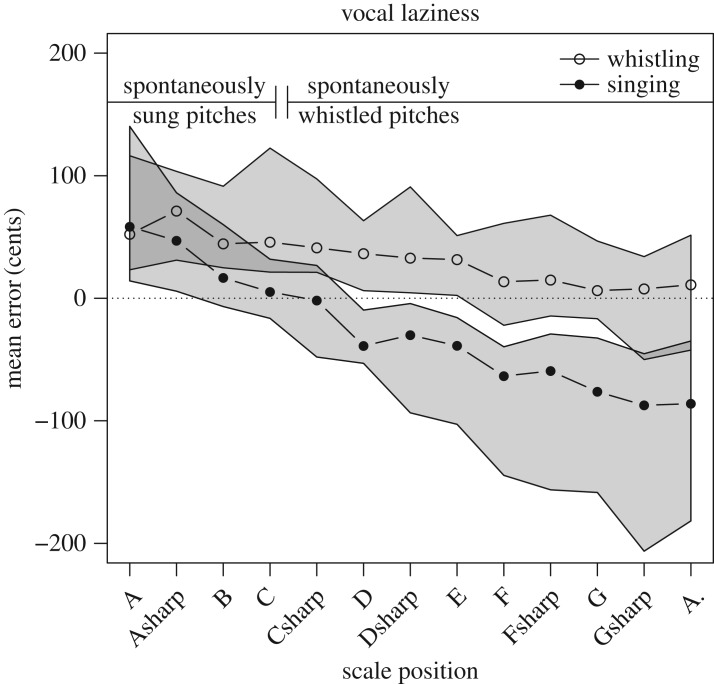
Vocal laziness. Median imitation inaccuracy is plotted for each stimulus note following the chromatic scale. Singing for males, singing for females, and whistling were performed in task appropriate octaves (starting from A2, A3 and A5, respectively), and are displayed on a common scale on this plot to facilitate comparison. The grey area encloses the interquartile range. The dotted line indicates a maximally accurate imitation score. Positive scores indicate imitations that were sharp and negative scores indicate imitations that were flat. Solid lines in the top portion of the figure indicate the ranges of participants' spontaneous singing and whistling pitches. Participant's spontaneous pitches tended to occupy the lower end of the stimulus range for singing and the upper end of the stimulus range for whistling. For both modes of production, accuracy was best for notes within the range of spontaneous pitches. Hence, while participants sung flat on average, they did not consistently transpose melodies downwards. Rather, participants shifted sung notes towards a point within the range of habitually produced pitches. This point may reflect a configuration of least vocal effort, distance from which may make pitch production more effortful. Vocal laziness, or a conservation of vocal effort, may lead singers to compress sung notes towards the pitch associated with a default or preferred configuration of the larynx.

In order to explore the possible causes of the flatness of singing and sharpness of whistling, we conducted a *post hoc* test of imitation inaccuracy as a function of the target pitch. Figure 4 plots mean imitation errors of whistling and singing for each target note. As before we observed consistently sharper scores for whistling than singing (*F*_1,643.4_ = 6.53, *p* < 0.05, standardized effect = 85.8, 95% CI = 72.8 to 98.5). We also observed a strong tendency for imitations to become more flat as the pitch height of target notes increased (*F*_1,642.8_ = 104.0, *p* < 0.05, standardized effect = −69.1, 95% CI = −83.1 to −55.2), and an interaction indicating that this effect was stronger for singing than for whistling (*F*_1,643.4_ = 14.4, *p* < 0.05, standardized effect = 51.5, 95% CI = 23.7 to 78.9). Figure 4 makes plain that high notes were sung flat, whereas low notes were sung sharp. This suggests that sung imitations may be compressed towards a particular point within a singer's vocal range.

### 3.3. Musical training

Each LMM was refitted with additional linear predictors for musical experience measured in years of formal training and the sex of the participant, excluding two participants who had prior training as vocalists. Neither musical experience nor sex significantly predicted any outcome measure. Parameter estimates for task and MBEA predictors were similar to those reported above, although with broader CIs that presumably reflect lost residual degrees of freedom from modelling a smaller sample with more predictors (see electronic supplementary material, file 3).

## 4. Discussion

The current experiment aimed to test the relative proficiency of human singing and whistling in the light of previous indications that although humans are the most proficient vocal learners among primates they may none-the-less have relatively coarse control over the laryngeal muscles that regulate vocal-pitch. We observed that participants tended to sing flat but whistle sharp. Singing and whistling imprecision were highly correlated, suggesting that some common mechanisms may contribute to errorfulness in both domains. Singing was also systematically less precise than whistling, suggesting a differential error proneness for the laryngeal and oral muscles, respectively. Since singing and whistling probe muscles that control the laryngeal sound source and the vocal-tract filter, respectively, they provide an avenue to study the motor control of both sound source and filter using a common metric.

Previous research has observed that trained singers are less precise at matching pitches with their voices than instrumentalists are at matching pitches with their instruments [51,55,56]. Although the violin produces a continuous range of pitches and is, therefore, capable of erring to the same degree as the voice, design features of the violin may give instrumentalists an advantage. For instance, the strings of the violin are tuned to ensure that a given placement of the bow will produce a reliable pitch. By contrast, pitch production by the larynx depends on complex and nonlinear interactions between multiple laryngeal muscles [76–82], the configuration of the articulatory muscles [19,83] and the action of the lungs [84–86], within a biological frame whose tuning may change as it matures and senesces.

We observed that pitch production by singing is also imprecise compared to pitch production by whistling. Although less is known about the bioacoustics of whistling relative to singing, both rely on the careful configuration of complex and interacting muscle groups within the vocal tract. Moreover, even people who seldom sing have more extensive experience controlling vocal-pitch than a whistled pitch through daily experience with speech. Speech involves a constant regulation of voice onsets and vocal-pitch height. Speakers use these cues to encode voiced compared to voiceless phonemes [33,34], the tones of a tonal language [35], stress on particular syllables [36], emphasis on certain words [37], the intonation of sentences to contrast declarative and interrogative modes [38] and to express a broad range of genuine or feigned emotions [39–43]. Sung pitches remain imprecise despite a lifetime of daily voice experience, suggesting that there are fundamental limitations on the precision of human vocal-pitch control at the level of the laryngeal muscles or the neuro-motor system that controls them [87–89].

### 4.1. Music and language share a voice in song and speech

Music and language are two frameworks that humans use to interact and communicate with one another. They are not exclusive to any one mode of production; for example, music can be expressed by blowing in some instruments or by banging on others and language can be expressed by writing or by making signs. Music and language share the use of the voice when they are expressed as singing and speaking, respectively.

The use of vocal-pitch as both the carrier for melody in song and for providing prosodic and phonetic cues in speech reflects part of a broader framework linking the evolution of musical and linguistic abilities in humans [90–92]. Many features are shared between language and music, such as processing sequences of sound over time [93–97], interpreting their meaning within the broader context of a musical or linguistic phrase [98–102], syntactic ordering of events [103–105], pacing of rhythmic movements [106–108] and vocal production learning [109–112]. The evolution of any of these abilities, including vocal production learning, may have been driven by selective pressures that predate singing or speaking, though they support both of these behaviours [3,96,113–115].

This shared history of selective pressures not specific to music is consistent with an existing view that the development of human musical scales may have been constrained to accommodate the imprecision of the voice [51]. The music of most cultures is built on scales containing a small number of degrees [116], leading most note categories to be separated by a full tone (200 cents). Scales of this construction may have allowed even novice singers to sing notes that were closer to being in tune than out of tune, most of the time.

### 4.2. The relatively recent evolution of the vocal-motor system

The greater precision of human orofacial-pitch control in whistling, relative to laryngeal-pitch control in singing, is consistent with a relatively recent evolution of the neuro-motor system that controls the laryngeal muscles [87]. Although many species can volitionally produce their species typical calls, few species have the capacity to add new calls to their repertoire through imitation. This ability is found in three lineages of songbird [3,117] and several lineages of mammal, including cetaceans [4–6], pinnipeds [7–11], bats [12,13] and possibly elephants [14,15]. Humans are notable as the only primate with a strong capacity for vocal imitation.

Non-human apes have been observed to produce a variety of novel sounds, but these are most often in the form of oral sounds, such as a ‘raspberry’ or a whistle that use the lips or tongue as a sound source [25–27,118], although these species may also have a limited degree of flexibility at the laryngeal sound source [22–24]. The most well-documented case is that of Koko the encultured Gorilla. Koko learned an extensive repertoire of novel sounds that she used primarily during play [23]. These sounds demonstrated a considerable degree of control over the muscles of articulation and respiration, but little of Koko's vocabulary involved voice production from the laryngeal sound source, suggesting a more limited degree of control over the laryngeal sound source than the rest of the vocal tract.

Sound imitation is a complex behaviour that engages a broad network of brain areas. Neuroimaging research in which human participants imitated articulatory patterns [119–122] or pitch patterns [123,124] have observed activation in brain areas related to motor-planning and execution, including the inferior frontal gyrus, anterior cingulate cortex, supplementary motor area, basal ganglia, cerebellum, primary somatosensory cortex and primary-motor cortex. Much of this network is conserved across primates [63,65,125–129] and contains somatotopic maps with separate representations of the various muscles of the body [128,130–135], inviting a neuro-comparative analysis at the level of muscle groups.

While the larynx-motor area of monkeys is found in premotor cortex and has limited involvement in vocal behaviour [136–139], the human larynx-motor area is found in primary-motor cortex and has a clear involvement in regulating vocal behaviour [124,133,140–147]. Non-human great apes have an intermediate phenotype [128,148,149]. These brain areas have distinct cytoarchitectural profiles; the primary-motor cortex has a greater abundance of descending motor fibres than the premotor cortex [150,151]. Likewise, monkeys lack a direct connection between the larynx-motor cortex and the nucleus ambiguus, which is the brainstem-motor nucleus that controls the laryngeal muscles [66,67]. Apes have a sparse, but extant, direct pathway between these areas, that is slightly more abundant in humans [63,64]. Vocal behaviour driven by primary-motor cortex began to evolve before the divergence of humans from other apes, but was elaborated over human evolution. Several theorists have speculated that the emergence of this pathway may have been a prerequisite to the evolution of speech [105,152–155].

By contrast, the motor areas controlling the lips and tongue are found in similar cytoarchitectural zones across primates [65,128]. Likewise, motor fibres descending to the facial nucleus, which is the brainstem nucleus that controls the lips and tongue, are more abundant than the equivalent pathway for the larynx [63–65,149]. This abundance of orofacial-motor fibres is common to monkeys, non-human apes and humans, suggesting an evolutionary history that predates the divergence of these clades.

This comparative analysis of both vocal-learning ability and its underlying neurophysiology suggests that volitional control over the orofacial muscles evolved earlier in the primate lineage than volitional control over the laryngeal muscles; whereas orofacial-motor control is evident in all primates, volitional control over the laryngeal muscles is lacking in monkeys, incipient in non-human apes and most evident humans.

Although human vocal-motor abilities are elaborated beyond the poorer vocal-motor abilities of other primates, the relatively imprecise control of pitch by the larynx may have been sufficient to satisfy the selective pressures for which it evolved. Singing may impose demands on vocal-pitch control beyond the scope for which this ability evolved.

### 4.3. Imprecise pitch imitation as the accumulation of neuro-motor noise

**Figure 5.**
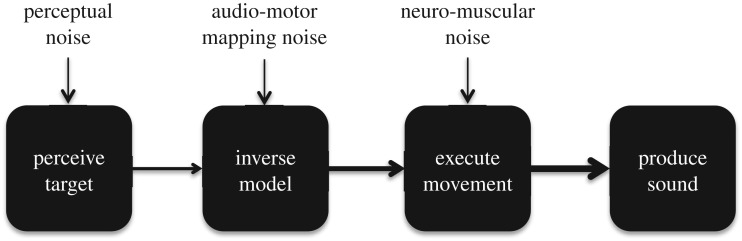
Propagation of error model. Audio-motor imitation requires singers to listen to a target melody, compute an inverse model that maps the target melody onto a sequence of movements that will reproduce it, then execute those movements. We posit that computational noise at each stage of this process may be propagated to subsequent stages. Perceiving target melodies probably engages similar processes within the auditory system for both singing and whistling, and computational noise in this system may explain the high degree of correlation between singing and whistling imprecision. Executing movements probably engages muscle-specific domains within the motor system and may explain why levels of imprecision differed for melodies sung with the larynx versus whistled with the tongue.

We observed a strong relationship between pitch perception abilities and imprecision in audio-motor imitation for both singing and whistling. Audio-motor imitation requires singers to listen to a target melody, compute an inverse model that maps the target melody onto a sequence of movements that would reproduce it, and finally, execute those movements. We propose that computational noise at each stage of this process may be propagated to subsequent stages, such that imitation errors are the accumulation of errors at each stage of processing (figure 5).

Perceiving target melodies probably engages similar processes within the auditory system for both singing and whistling, and computational noise in perceiving pitch targets and retaining them in memory may explain the high degree of correlation between singing and whistling imprecision. Executing movements for sound production probably engages muscle-specific domains within the motor system from primary-motor cortex through descending corticobulbar pathways. The somatotopic organization of motor cortex by muscle effector [128,133,144,156] may cause neuro-motor noise for laryngeal movements during singing to be independent from the neuro-motor noise for tongue movements during whistling.

From analogy with songbirds, which are the most extensively studied animal model of vocal learning and imitation, inverse models that map target pitches onto motor commands appear to be computed by a thalamo-cortico-striatal loop [157,158]. Non-invasive brain imaging studies in humans have begun to support this analogy [121,122,124]. The parts of the thalamus, striatum and cortex that are relevant to movement are somatotopically organized into populations that control different groups of muscles [130,132,133,159]. Hence, it seems possible that separate but parallel neural networks compute inverse models for the larynx and the tongue, although further research is needed to assess the separation of inverse-model processes in the striatum for different muscle groups. Indeed, the sensorimotor computations of the inverse model have been proposed to be a key deficit in poor-pitch singing [47].

The movement outcomes of motor commands are assessed by a cerebro-cerebellar forward model network that compares the intended motor command to feedback from the sensory periphery [160–162]. Although the forward model was conceived as a mechanism that corrects motor commands based on proprioceptive feedback, it may also use auditory feedback for movements that produce a sound [163]. Since the cerebellum also contains somatotopic representations of the body [164], neuronal noise in forward model processes may also contribute to effector-specific imprecision.

We also observed a correlation between singing and whistling precision abilities after controlling for the mutual influence of perceptual abilities. There may be additional factors that have a mutual influence on singing and whistling production. One candidate is the mutual influence of respiratory motor-control, as expiration provides the mechanical drive for both singing and whistling. For both modes of sound production, increases in sound pressure level are related to higher frequencies [165,166]. Hence, fluctuations in expiatory flow may translate into unstable singing and whistling. Computational noise from shared processes such as respiration, together with independent and effector-specific noise in motor execution processes, may explain the strong correlation between singing and whistling imprecision with a consistent shift towards lower levels of precision in singing.

### 4.4. Vocal laziness

We observed no correlation between singing and whistling accuracy after controlling for perceptual ability, but instead observed a consistent bias to sing flat. This replicated a previous finding that untrained singers tend to compress pitches towards a habitual range [44]. From these exploratory analyses, we hypothesize that each individual's larynx may have a preferred frequency that it produces in a default configuration. Pitches produced at a participant's most accurate note may require the least muscular effort, while pitches further from this preferred note, in either direction, may require greater muscular effort.

The cricothyroid (CT) and thyroarytenoid (TA) muscles are the primary regulators of vocal-pitch in mammals. Contraction of the CT muscle rocks the thyroid cartilage forward, thereby stretching and increasing the tension of the vocal folds, and causing them to vibrate at a higher fundamental frequency (*F*_0_) [76,78–80,167]. The TA muscle may relax the vocal folds, and in that sense acts as an antagonist to the CT muscle to decrease *F*_0_. However, the role of the TA muscle is complicated by strong interactions with the state of the CT muscle [86,168,169].

Electromyographic studies have not examined the muscular profiles of pitch levels above compared to below participant specific habitual levels. However, as *F*_0_ decreases to the level where the CT muscle is no longer active, other laryngeal muscles may become engaged [80]. A different but equally active process may be engaged when singers lower *F*_0_ from the habitual level compared to raising it above the habitual level. An active process for producing lower than habitual pitches may explain why participants tended to sing sharp within this range because erring towards a habitual pitch may be less effortful than erring away from it. Vocal laziness, or a conservation of vocal effort, may lead singers to compress sung notes towards the pitch associated with a default or preferred configuration of the larynx.

## 5. Conclusion

We report the results of a study on pitch imitation in singing with the laryngeal sound source compared with whistling with an oral sound source. Sung imitations were less precise than whistled imitations, although neither were on the order of precision that have previously been reported for continuous pitch instruments, such as the violin. While biological pitch production in general may be less reliable than instrumental pitch production, the neuro-motor control of the larynx for pitch production is particularly coarse. From the relatively recent evolution of vocal production learning in great apes, we suggest that evolution has not tuned the human vocal-motor system to the same degree as other neuro-muscular systems.

## Supplementary Material

Sung and whistled pitch imitation: Supplementary analyses.

## Supplementary Material

Vocal laziness by sex: Supplementary figures

## Data accessibility

The raw data, processed data and experimental materials are archived in Dryad Digital Repository: http://dx.doi.org/10.5061/dryad.504t7 [69].
