# Supplementary material for: Poor neuro-motor tuning of the human larynx: a comparison of sung and whistled pitch imitation
Source: R Soc Open Sci. 2018 Apr 18;5(4):171544. doi: 10.1098/rsos.171544 (PMC5936900; doi:10.1098/rsos.171544)
Supplement: Sung and whistled pitch imitation: Supplementary analyses. [file rsos171544supp1.docx]

Sung and whistled pitch imitation: Supplementary Analyses

Belyk

1/8/2018

**Musical training did not significantly predict any of our dependent variables. For one measure (interval imprecision) adding the additional predictor for musical experienced rendered a previously significant effect of task (whistling vs. singing) insignificant. This change is the result of wider confidence intervals from lost degrees of freedom (due to removing two participants and fitting extra parameters) rather than to any change in the model estimates.**

**Note Imprecision**

## Analysis of Deviance Table (Type III Wald F tests with Kenward-Roger df)
##
## Response: dv
## F Df Df.res Pr(>F)
## (Intercept) 43.6981 1 21.263 1.425e-06 ***
## task 12.8692 1 22.327 0.0016123 **
## MBEA 21.2521 1 20.781 0.0001549 ***
## lessons_years 3.1823 1 19.651 0.0898910 .
## sex 0.9782 1 19.919 0.3345169
## ---
## Signif. codes: 0 '***' 0.001 '**' 0.01 '*' 0.05 '.' 0.1 ' ' 1

Confidence Intervals

## 2.5 % 97.5 %
## .sig01 15.151279 44.4583789
## .sigma 17.797022 33.7465011
## (Intercept) 387.301118 717.6647588
## taskWhistle -44.539131 -13.3653385
## MBEA -634.550145 -257.4759401
## lessons_years -6.136086 0.3842005
## sexmale -45.424699 12.0942984

**Interval Imprecision**

## Analysis of Deviance Table (Type III Wald F tests with Kenward-Roger df)
##
## Response: dv
## F Df Df.res Pr(>F)
## (Intercept) 31.3582 1 20.838 1.518e-05 ***
## task 4.1997 1 22.202 0.0524293 .
## MBEA 16.2163 1 20.528 0.0006325 ***
## lessons_years 1.7535 1 19.783 0.2005277
## sex 0.1403 1 19.959 0.7119601
## ---
## Signif. codes: 0 '***' 0.001 '**' 0.01 '*' 0.05 '.' 0.1 ' ' 1

Confidence Intervals

## Computing bootstrap confidence intervals ...

## 2.5 % 97.5 %
## .sig01 26.505695 58.2943286
## .sigma 17.815775 32.5858802
## (Intercept) 382.274572 802.0067377
## taskWhistle -30.040241 -0.7178481
## MBEA -735.535230 -248.9699768
## lessons_years -7.523497 1.5811346
## sexmale -50.521138 33.0065376

**Note Inaccuracy**

## Analysis of Deviance Table (Type III Wald F tests with Kenward-Roger df)
##
## Response: dv
## F Df Df.res Pr(>F)
## (Intercept) 3.3475 1 21.890 0.08096 .
## task 23.6618 1 22.668 6.779e-05 ***
## MBEA 2.3007 1 21.030 0.14420
## lessons_years 0.4956 1 19.308 0.48986
## sex 1.1163 1 19.726 0.30349
## ---
## Signif. codes: 0 '***' 0.001 '**' 0.01 '*' 0.05 '.' 0.1 ' ' 1

Confidence Intervals

## Computing bootstrap confidence intervals ...

## 2.5 % 97.5 %
## .sig01 3.914217e-09 57.182920
## .sigma 5.051414e+01 87.430024
## (Intercept) -4.985013e+02 38.253847
## taskWhistle 6.186532e+01 150.736690
## MBEA -8.895705e+01 519.729728
## lessons_years -7.018647e+00 3.502106
## sexmale -7.703283e+01 20.986144

**Interval Inaccuracy**

## Analysis of Deviance Table (Type III Wald F tests with Kenward-Roger df)
##
## Response: dv
## F Df Df.res Pr(>F)
## (Intercept) 0.4905 1 21.582 0.491191
## task 8.0290 1 22.447 0.009552 **
## MBEA 0.1374 1 20.953 0.714614
## lessons_years 0.1259 1 19.529 0.726547
## sex 0.4281 1 19.868 0.520432
## ---
## Signif. codes: 0 '***' 0.001 '**' 0.01 '*' 0.05 '.' 0.1 ' ' 1

Confidence Intervals

## Computing bootstrap confidence intervals ...

## 2.5 % 97.5 %
## .sig01 1.096309e-06 14.995874
## .sigma 7.850018e+00 14.633171
## (Intercept) -8.054276e+01 35.332355
## taskWhistle 3.062038e+00 16.465689
## MBEA -5.100029e+01 80.409014
## lessons_years -9.967925e-01 1.505634
## sexmale -1.462513e+01 7.478296

| **ID** | **sex** | **age** | **lessons_binary** | **lessons_years** | **lessons_instr** |
| --- | --- | --- | --- | --- | --- |
| s01 | male | 21 | yes | 2 | drumset |
| s03 | female | 20 | yes | 4 | piano |
| s04 | female | 19 | yes | 15 | violin/piano/cello |
| s05 | female | 21 | yes | NA | piano |
| s07 | male | 20 | no | 0 | none |
| s08 | female | 22 | yes | 4 | guitar/piano |
| s09 | male | 27 | no | 0 | none |
| s10 | female | 26 | no | 0 | none |
| s13 | female | 21 | yes | 1.5 | piano |
| s14 | male | 23 | yes | 10 | cello |
| s15 | female | 22 | yes | 6 | piano |
| s16 | female | 20 | yes | 15 | vocals |
| s17 | female | 29 | yes | 2 | vocals |
| s18 | female | 22 | no | 0 | piano |
| s21 | female | 29 | yes | 11 | piano |
| s22 | female | 24 | yes | 6 | piano |
| s23 | female | 19 | yes | 6 | guitar |
| s25 | female | 18 | yes | 1 | piano |
| s26 | female | 19 | yes | 1 | guitar |
| s27 | male | NA | NA | NA | NA |
| s28 | female | 18 | yes | 9 | piano |
| s29 | male | 19 | yes | 1 | keyboard |
| s30 | female | 19 | yes | 4 | guitar |
| s31 | male | 21 | yes | 15 | cello |
| s32 | female | 20 | yes | 10 | guitar |
| s33 | female | 18 | yes | 7 | flute |
| s34 | female | 19 | yes | 5 | guitar |
| s35 | female | 20 | yes | 6 | violin |
