# Supplementary material for: Poor neuro-motor tuning of the human larynx: a comparison of sung and whistled pitch imitation
Source: R Soc Open Sci. 2018 Apr 18;5(4):171544. doi: 10.1098/rsos.171544 (PMC5936900; doi:10.1098/rsos.171544)
Supplement: Vocal laziness by sex: Supplementary figures [file rsos171544supp2.docx]

Figure 4 supplement 1: Singing and whistling inaccuracy plotted for females.

Figure 4 supplement 2: Singing and whistling inaccuracy plotted for males.
